# Invisible Smoke: Analyzing and Enhancing the Security of Smoke Detectors Under Intentional Electromagnetic Interference

**DOI:** 10.3390/s26154678

**Published:** 2026-07-23

**Authors:** Sheng Liu, Kai Liu, Chen Yan, Kaikai Pan, Xiaoyu Ji, Wenyuan Xu

**Affiliations:** College of Electrical Engineering, Zhejiang University, Hangzhou 310027, China; liu_sheng@zju.edu.cn (S.L.); liukai01@zju.edu.cn (K.L.); pankaikai@zju.edu.cn (K.P.); xji@zju.edu.cn (X.J.); wyxu@zju.edu.cn (W.X.)

**Keywords:** smoke detector, intentional electromagnetic interference, false alarm, hardware security, sensor spoofing

## Abstract

Smoke detectors are pivotal sensors in fire protection systems, serving as the primary trigger for alarm mechanisms and structural fire-response strategies. Their security is fundamental to the stable operation of the entire fire-protection infrastructure. This paper investigates the manipulation of commercial smoke detectors through the injection of Intentional Electromagnetic Interference (IEMI), which can induce false alarms or suppress alarms even in the presence of smoke. Our core insight is that, despite rigorous Electromagnetic Compatibility (EMC) testing, the button and amplification circuits in the detector architectures evaluated in this study can remain susceptible to coupling with IEMI attack signals. We validated these threats on nine gas sensors and seven off-the-shelf commercial smoke detectors, achieving an attack distance of over 3 m with a total power maintained within 50 W. We further observed similar IEMI-induced effects in the additional gas-detection devices evaluated in this study, including an alcohol detector, a toxic gas detector, and an air-quality detector. Finally, we propose both hardware- and software-based countermeasures to mitigate these risks.

## 1. Introduction

Smoke detectors have become an indispensable part of modern safety infrastructure [1]. In the residential sector, mandatory safety regulations have driven their penetration rate to approximately 96% in the United States. Moreover, the rapid evolution of the Internet of Things (IoT) has transformed these devices into interconnected nodes within smart building ecosystems [2]. For example, in industrial environments, smoke detectors are critical components integrated into automated fire suppression and Supervisory Control and Data Acquisition (SCADA) systems [3].

**Figure 1:**
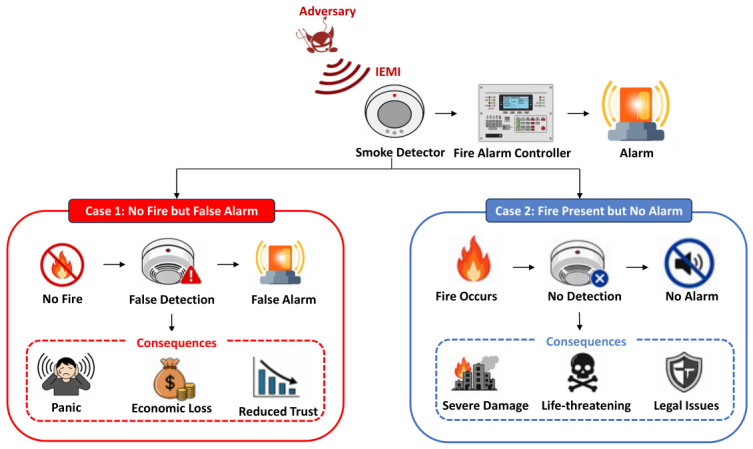
IEMI triggers false alarms or suppresses detection in smoke alarms, leading to severe consequences.

In this paper, we investigate the security of smoke-based fire protection systems and their underlying sensors. Our research is motivated by the stringent safety requirements of these systems, where any failure can lead to catastrophic consequences, particularly in sensitive environments such as medical or chemical laboratories. Incorrectly sensing smoke concentrations poses a significant threat to both life and property. False alarms can incur exorbitant costs, such as the accidental activation of sprinkler systems causing irreversible damage to electronic equipment [4], or the sudden alarm signals triggering mass panic in crowded areas [5]. Furthermore, repeated false alarms can induce a “cry wolf” effect, where occupants become desensitized and subsequently ignore genuine fire warnings. Conversely, a failure to detect smoke (false negatives) can cause occupants to miss the optimal window for evacuation, resulting in severe casualties, as illustrated in Figure 1.

Although smoke detectors play a critical role in fire protection systems, security research on smoke detectors remains limited. A prior study [6] has shown that carefully modulated laser signals can interfere with optical beam smoke detectors (OBSDs). However, OBSDs are typically deployed in large, open spaces such as factories and airports. For point-type smoke detectors, which are more commonly used in homes and buildings, this laser-based attack method is not applicable. Whether point-type smoke detectors possess new vulnerabilities and attack surfaces therefore remains an open question.

This study aims to systematically evaluate the vulnerability of smoke detectors under Intentional Electromagnetic Interference (IEMI), analyze the potential interference mechanisms and attack feasibility, and subsequently propose effective protection solutions to enhance the detectors’ electromagnetic immunity, ultimately improving the reliability and security of fire protection systems in complex electromagnetic environments. Achieving this goal involves addressing two primary challenges:

- Obscure Internal Mechanisms: Commercial smoke detectors are typically designed as closed, low-cost, and application-specific devices. Their internal circuits, amplifier structures, alarm decision logic, and anti-interference designs are generally not publicly disclosed. As a result, attackers can hardly obtain complete circuit models, making it difficult to theoretically predict how IEMI signals affect the sensing chain with high precision.
- Regulated Electromagnetic Resilience: According to the EN 50130-4 standard [7], smoke detectors are typically required to undergo rigorous Electromagnetic Compatibility (EMC) testing. They usually incorporate interference-resistant designs, such as electromagnetic shielding and optimized PCB layouts. Under electromagnetic interference, they are expected not to generate false alarms or incorrect alarm responses, while maintaining the level of smoke sensitivity.

To address these challenges, we first systematically analyzed the internal structure of smoke detectors by disassembling commercial devices and referring to relevant design reports [8]. Based on this analysis, we infer that the amplifier and button circuits inside the device may serve as potential coupling entry points for IEMI signals.

Next, we conducted both simulations and physical experiments on the amplifier circuit and the button circuit, validating that carefully designed modulated signals can interfere with these two components. Based on this finding, we can cause the smoke detector to trigger false alarms or suppress alarms even in the presence of smoke.

To validate the threats posed by this vulnerability, we evaluated it on nine sensor modules and seven commercial smoke detectors. Unless otherwise stated, the experimental findings and conclusions reported in this paper refer to the commercial devices and detector architectures evaluated under the laboratory conditions described in this study. We further examined the threat under different attack positions, angles, and obstacle-blocking conditions. In addition, we found that this vulnerability also exists in other commercial gas detection devices, potentially causing severe consequences such as undetected toxic leaks, catastrophic explosions, and costly false alarms that disrupt industrial operations.

To address the security threats posed by IEMI signals, we analyze the underlying mechanism by which IEMI signals affect the internal circuitry. In addition to conventional software- and hardware-based protection methods, we propose a detection method based on the observation that IEMI-induced signals and real smoke signals exhibit different data features. This method can help reduce both false alarms and alarm suppression, and may provide useful guidance for future smoke detector design and the standardization of alarm-triggering logic. In summary, our contributions are as follows:

- We systematically analyze the circuit structure and operating logic of commercial smoke detectors, characterize the physical-level vulnerabilities of their amplifier and button circuits when exposed to IEMI, and discuss the potential threats caused by these vulnerabilities.
- To evaluate the severity of this vulnerability, we design *Invisible Smoke*, an attack that can trigger false alarms in smoke detectors or, more critically, suppress alarms even in the presence of smoke. We validate this attack on both sensor modules and commercial smoke detectors, and further show that the threat can be generalized to other gas-detection devices.
- We propose and evaluate hardware- and software-level mitigation measures, including an attack signal detection method based on false sensor data features, to address the threats posed by such vulnerabilities.

## 2. Background

### 2.1. Fire Alarm Systems and Smoke Detectors

A fire protection system [9] is primarily composed of three components: smoke detectors, a fire alarm controller, and response mechanisms (e.g., notification appliances and sprinkler systems). It works through a simple three-step process. First, smoke detectors sense a fire and send a signal to the fire alarm controller. The controller then confirms the alarm and activates the corresponding emergency devices, such as strobes, sirens, and sprinkler systems, to warn occupants to evacuate and help suppress the fire.

Smoke detectors are key front-end sensing devices in fire alarm systems. According to their detection mechanisms and installation forms, common smoke detectors can be divided into point-type smoke detectors and optical beam smoke detectors (OBSDs). An OBSD typically consists of a transmitter and a receiver, and it determines the presence of smoke by monitoring the attenuation of light intensity along a long optical path. Therefore, OBSDs are suitable for large or high-ceiling spaces, such as warehouses, factories, atriums, and exhibition halls. In contrast, point-type smoke detectors are more flexible to install and have lower cost. They can be deployed in a wider range of daily scenarios, such as residences, offices, corridors, hotels, and schools. Therefore, point-type smoke detectors currently have broader application scenarios.

Conventional point-type smoke detectors can be mainly divided into two categories: ionization smoke detectors [10] and photoelectric smoke detectors [11]. Ionization smoke detectors use a small amount of radioactive material to ionize the air and determine the occurrence of a fire by detecting the change in current caused by smoke particles entering the ionization chamber. Photoelectric smoke detectors use a light source and a photoelectric sensor to detect light scattering or light intensity variations caused by smoke particles. Since photoelectric smoke detectors perform well in detecting smoldering residential fires and have a lower rate of cooking-related false alarms than ionization detectors, they have become more widely used in modern residential applications. Therefore, this work focuses on the security of widely deployed point-type photoelectric smoke detectors.

### 2.2. Working Principle of Photoelectric Smoke Detectors

**Figure 2:**
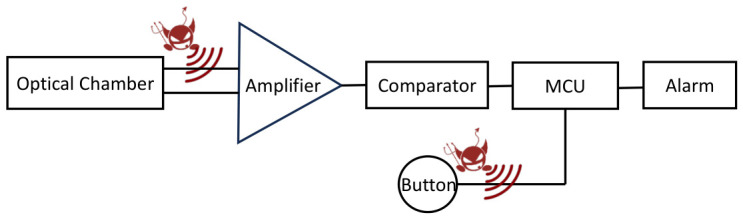
An illustration of the general signal-conditioning path of a smoke detector and the attack surface of EMI.

Photoelectric smoke detectors, also known as optical smoke detectors, operate primarily on the principle of light scattering [8]. This method is particularly effective at detecting large-particle smoke generated by smoldering fires. The overall workflow and triggering process of photoelectric smoke detectors are shown in Figure 2.

#### 2.2.1. Optical Chamber Design

**Figure 3:**
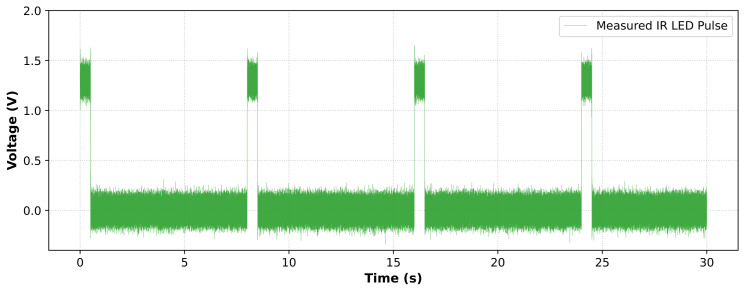
Periodic pulsed signal emission from the IR-LED in the optical sensing chamber.

The core of the detector is an optical sensing chamber, often referred to as an “optical maze.” This structure is designed to baffle external ambient light while allowing smoke particles to circulate freely. Inside the chamber, an infrared light-emitting diode (IR-LED) and a photodiode are positioned at an oblique angle, typically between $90^{\circ }$ and $135^{\circ }$, such that they do not have a direct line of sight. The IR-LED emits pulsed signals periodically rather than continuous light to reduce power consumption and improve the signal-to-noise ratio, as shown in Figure 3.

#### 2.2.2. Light Scattering Physics

The detection mechanism switches between two states based on the presence of particulate matter:

1. Clear Air State: In the absence of smoke, the light beam is completely absorbed by the light trap. The photodiode remains in a quiescent state, generating only a negligible dark current:
  (1) $$I_{\mathrm{sensor}}=I_{\mathrm{dark}}\approx 0.$$
2. Smoke Detection State: The entry of smoke particles induces light scattering, generating a specific scattered light intensity $I_{s}$ as an optical quantity. The photodiode receives this scattered light and converts it into a photocurrent $I_{p}$ as an electrical quantity. The direct conversion relationship between the two is determined by the optical collection efficiency of the sensing chamber $\eta_{\mathrm{opt}}$, the effective photosensitive area of the photodiode $A_{\mathrm{PD}}$, and its spectral responsivity $R_{\lambda }$, which can be concisely expressed as:
  (2) $$I_{p}=R_{\lambda }\eta_{\mathrm{opt}}A_{\mathrm{PD}}I_{s}.$$

#### 2.2.3. Signal Processing and Alarm Trigger

The photodiode converts the received photons into a photocurrent $I_{p}$. This current is amplified and converted into a voltage signal $V_{\mathrm{sig}}$ using a transimpedance amplifier. Subsequently, an analog-to-digital converter (ADC) or a hardware comparator is employed to evaluate the signal. The device logic triggers an alarm when the signal exceeds a predefined threshold:

(3) $$V_{\mathrm{sig}}=G\cdot I_{p}>V_{\mathrm{threshold}},$$

where *G* represents the gain of the transimpedance amplifier. This threshold-based system, implemented via a comparator circuit, ensures that minor fluctuations, such as occasional dust particles, do not cause false positives.

#### 2.2.4. Test and Silence Logic

Most smoke detectors incorporate a physical button for manual testing and alarm silencing. The device controller interprets the button event according to the current operating state and executes different logic.

- Manual Self-Test: When the button is pressed in the standby state, the device performs a self-test without requiring real smoke. This process is used to verify that key components, such as the electronic circuitry, audible sounder, and battery, are operational and capable of triggering an alarm.
- Alarm Silencing (Hush Mode): If the button is pressed during an active nuisance alarm, such as an alarm caused by cooking smoke, the detector is temporarily silenced. During this fixed duration, the device is not completely disabled. If the smoke concentration continues to increase to a critical level, the alarm will override the hush mode and sound again.

## 3. Threat Model

This work studies the vulnerability of the analog sensing circuitry that drives commercial smoke detectors, and the effect of induced electromagnetic noise on internal amplifier components and buttons to reveal a widespread vulnerability. By exploiting these vulnerabilities, an attacker can manipulate the internal signal voltage of the victim’s smoke detectors, causing either false alarms or alarm suppression. Specifically, the attacker can artificially increase the sensing signal to trigger a false alarm, or decrease the sensing signal to prevent the detector from alarming even in the presence of smoke. We assume that the attacks have the following goals and capabilities:

Attack Goal. The attacker’s goal is to covertly trigger false alarms or prevent alarms from being raised in the presence of real fires. In this work, we focus on attacks against a single smoke detector rather than coordinated attacks on multiple detectors.

Non-contact Access. We assume the attacker can approach the target smoke detectors within a few meters, but she cannot physically touch or damage them due to safety and stealthiness concerns. Alternatively, the adversary can leave a camouflaged EMI device nearby and control it remotely.

Prior Knowledge. We assume that adversaries possess prior knowledge of the target devices. Since many smoke detectors are commercial off-the-shelf (COTS) products readily available on the market, the adversary could acquire a replicate device of the same model to conduct preliminary tests. Furthermore, in practice, fire alarm installations within a specific region often employ identical detector models.

## 4. Feasibility and Vulnerability Investigation

**Figure 4:**
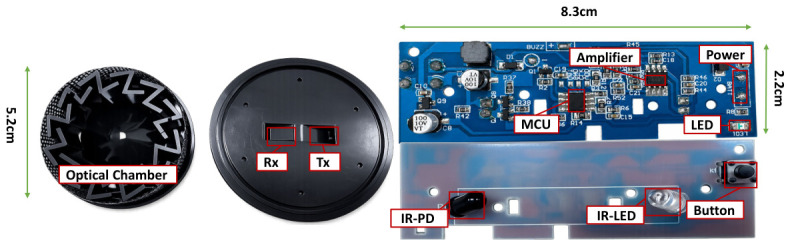
Internal circuit structure of the KKQ-718 commercial smoke detector.

In this section, we investigate how IEMI affects the internal components of smoke detectors and causes false alarms or alarm suppression through theoretical analysis and feasibility experiments. By reverse-engineering seven commercial smoke detectors, we discovered that their circuits share many similar structural characteristics, as illustrated in Figure 4. Generally, there are two primary methods to trigger a smoke alarm: the first involves the actual detection of smoke, which induces a series of signal variations within the sensing circuit; the second is a manual trigger via the test/reset button. Since the underlying physical principles of these two methods differ significantly, we will investigate their respective triggering mechanisms separately.

### 4.1. The Impact of IEMI Signals on Smoke-Sensing Circuitry

Our analysis shows that the smoke sensing circuit mainly consists of a sensing element, an amplifier, a comparator, a Microcontroller Unit (MCU), and an alarm module. The analog front-end may become a sensitive entry point for IEMI. External IEMI can couple into the amplifier front-end through sensor leads, PCB traces, power lines, parasitic capacitance, or enclosure gaps. It should also be noted that IEMI can enter the operational amplifier through its power-supply pins, because practical op-amps have finite and frequency-dependent power-supply rejection. Once the interference enters the amplifier, it may alter the amplifier output and affect the subsequent decision-making stage. In the following section, we analyze how IEMI signals affect the amplifier.

For an ideal operational amplifier, the input–output relationship is assumed to be linear. Therefore, the output voltage is proportional to the input voltage and can be expressed as

(4) $$v_{\mathrm{out}}\left(t\right)=Av_{\mathrm{in}}\left(t\right)$$

where *A* is the voltage gain of the amplifier. However, in practical operational amplifier circuits, an op-amp is not simply an ideal linear gain block. A typical operational amplifier usually consists of three functional stages: the differential input stage, the voltage gain stage, and the output stage. In a practical operational amplifier, several circuit modules may exhibit nonlinear behavior. In particular, the dominant nonlinearities are mainly associated with the P-N junctions in Bipolar Junction Transistor (BJT) devices, such as the base-emitter and base-collector junctions of the input transistors. These junctions are closely located at the amplifier front-end and can respond asymmetrically to coupled high-frequency IEMI signals, thereby converting part of the interference into a DC component.

The rectification effect originates from the nonlinear input–output characteristic of the P-N junction. The junction current can be described by the Shockley diode equation:

(5) $$i_{d}\left(t\right)=I_{0}\left(e^{\frac{v_{d}\left(t\right)}{nV_{T}}}-1\right),$$

where $i_{d}\left(t\right)$ is the junction current, $v_{d}\left(t\right)$ is the voltage across the junction, $I_{0}$ denotes the reverse saturation current of the P-N junction, *n* is the ideality factor, and $V_{T}$ is the thermal voltage. Due to this exponential nonlinearity, a coupled high-frequency signal can be partially converted into a DC component through the nonlinear response of the P-N junction.

**Figure 5:**
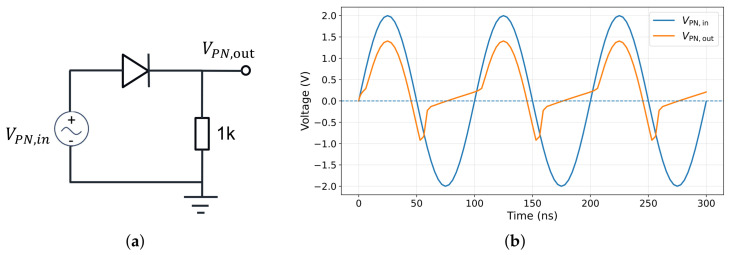
Multisim−based verification of the rectification effect in a P−N junction circuit: (**a**) simulation circuit and (**b**) simulated rectified output waveform.

To verify this mechanism, we designed a simple simulation circuit in Multisim. As shown in Figure 5, the high-frequency input signal is rectified by the nonlinear P-N junction, resulting in a DC component at the output. The remaining high-frequency components are attenuated by the circuit bandwidth, RC networks, and parasitic capacitances, while the DC component is preserved and amplified, eventually appearing as a stable DC offset.

**Figure 6:**
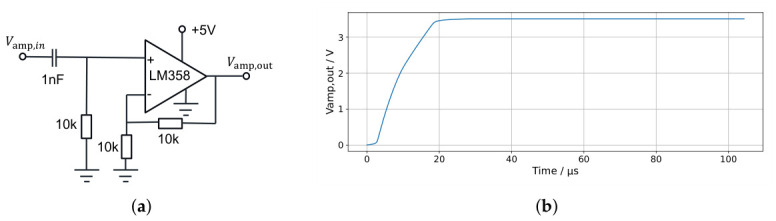
Multisim−based verification of the rectification effect in an LM358 amplifier circuit: (**a**) simulation circuit and (**b**) simulated rectified output waveform.

Similarly, we designed an LM358-based verification circuit in the simulation environment, as shown in Figure 6. In the simulation, a 10 MHz sinusoidal signal with an amplitude of 2 V was applied to the amplifier input. The output undergoes a short transient rising stage and then settles to a stable DC offset, verifying the rectification effect induced by the nonlinear amplifier circuit.

**Figure 7:**
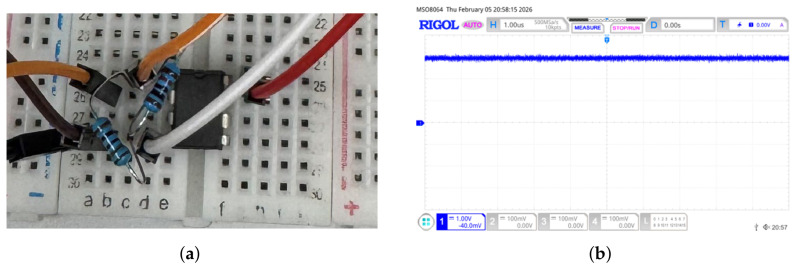
Experimental setup of the amplifier−based verification circuit and the stable DC bias trace observed on the oscilloscope. (**a**) Experimental setup for wired injection on the amplifier. (**b**) DC offset of $V_{\mathrm{amp},\mathrm{out}}$ observed on the oscilloscope.

In the physical experiment, we built an LM358-based op-amp circuit on a breadboard to validate this effect. The corresponding experimental setup is shown in Figure 7a. By injecting IEMI signals at specific resonant frequencies, such as 198 MHz, a significant and controllable DC offset was induced at the output, as shown in Figure 7b.

Building upon the amplification stage, the processed analog signal—whether it originates from a legitimate sensor reading or an IEMI-induced DC offset—is subsequently fed into a comparator. A comparator serves as a critical mixed-signal interface that bridges the analog and digital domains within the smoke detector’s circuitry.

The primary function of the comparator is to evaluate the amplified input voltage ($V_{in}$) against a predefined reference safety threshold ($V_{ref}$). The transfer characteristic of an ideal comparator can be modeled as a simple step function:

(6) $$V_{\mathrm{out}}=\left\{\begin{array}{cc} V_{\mathrm{oh}}, & \mathrm{if}\;V_{\mathrm{in}}\geq V_{\mathrm{ref}}\\ V_{\mathrm{ol}}, & \mathrm{if}\;V_{\mathrm{in}}<V_{\mathrm{ref}} \end{array}\right.$$

where $V_{oh}$ and $V_{ol}$ represent the high and low digital logic levels, respectively.

**Figure 8:**
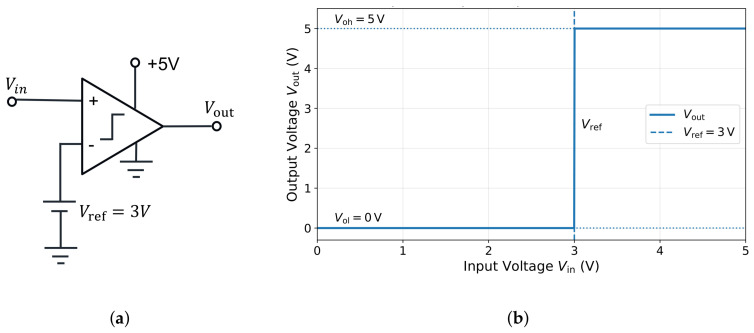
Performance analysis of the comparator: (**a**) circuit schematic; (**b**) observed transition between high and low levels.

In the context of the aforementioned IEMI vulnerability, the interplay between the amplifier and the comparator becomes the crux of the attack. If the adversary tunes the EMI signal such that the resulting amplified DC offset exceeds the reference threshold ($V_{in}\geq V_{ref}$), the comparator is deceived into outputting a digital ‘High’ signal ($V_{oh}$), as shown in Figure 8. Consequently, the subsequent MCU interprets this logic state as a genuine physical event, ultimately triggering a false alarm even in the complete absence of actual smoke.

To characterize the relationship among the incident electromagnetic frequency, the field-to-circuit coupling path, and the resulting amplifier output offset, we use a simplified empirical three-stage coupling–rectification model:

(7) $$\Delta V_{\mathrm{amp},\mathrm{out}}\left(f\right)=K_{r}\left(f\right){\left|G_{2}\left(f\right)G_{1}\left(f\right)E_{\mathrm{inc}}\left(f\right)\right|}^{2},$$

where $\Delta V_{\mathrm{amp},\mathrm{out}}\left(f\right)$ is the RF-induced DC offset at the amplifier output, *f* is the incident or injected RF frequency, and $E_{\mathrm{inc}}\left(f\right)$ is the incident electromagnetic field. The term $G_{1}\left(f\right)$ represents the frequency-dependent transfer function from the incident electromagnetic field to the RF voltage induced at the injection point [12]. This term empirically captures the space-to-circuit coupling effects associated with the sensor-equivalent leads, enclosure apertures, PCB or breadboard traces, parasitic capacitance, parasitic inductance, grounding structure, and field polarization. The term $G_{2}\left(f\right)$ represents the frequency-dependent transfer function from the injection point to the amplifier front end. It includes the effects of the sensor-equivalent path, PCB or breadboard traces, input bias network, input filtering components, protection devices, power-supply coupling, grounding path, parasitic impedances, and the frequency-dependent input impedance of the amplifier [13].

Thus, $G_{2}\left(f\right)G_{1}\left(f\right)E_{\mathrm{inc}}\left(f\right)$ represents the RF voltage reaching the nonlinear amplifier input stage. The coefficient $K_{r}\left(f\right)$ is a frequency-dependent equivalent RF-to-DC rectification coefficient [14]. It accounts for the effective nonlinear rectification efficiency at the amplifier front end, the low-frequency gain from the rectified component to the amplifier output, impedance-related scaling factors, and frequency-dependent rectification paths that are not explicitly separated in $G_{1}\left(f\right)$ and $G_{2}\left(f\right)$. In general, $K_{r}\left(f\right)$ is treated as a real-valued and potentially signed empirical coefficient. Since the term ${\left|G_{2}\left(f\right)G_{1}\left(f\right)E_{\mathrm{inc}}\left(f\right)\right|}^{2}$ is nonnegative, the polarity of the RF-induced DC offset is determined by the sign of $K_{r}\left(f\right)$. Therefore, a change in the dominant rectification path or effective nonlinear response with frequency can lead to different offset polarities at different RF frequencies. Under weak nonlinear operation, the rectified DC component is approximately proportional to the squared RF voltage at the amplifier input. Therefore, the output DC offset is modeled as proportional to ${\left|G_{2}\left(f\right)G_{1}\left(f\right)E_{\mathrm{inc}}\left(f\right)\right|}^{2}$, with the frequency-dependent coefficient $K_{r}\left(f\right)$ describing the magnitude and polarity of the equivalent RF-to-DC conversion.

This model explicitly describes three physical stages: the coupling of the incident electromagnetic field to the injection point through $G_{1}\left(f\right)$, the RF transfer from the injection point to the amplifier front end through $G_{2}\left(f\right)$, and the nonlinear RF-to-DC conversion at the amplifier front end through $K_{r}\left(f\right)$. Therefore, even under the same incident field strength or equivalent injection condition, different frequencies can produce different output offsets because $G_{1}\left(f\right)$, $G_{2}\left(f\right)$, and $K_{r}\left(f\right)$ are frequency-dependent. In particular, $G_{1}\left(f\right)$ and $G_{2}\left(f\right)$ determine the RF voltage magnitude reaching the nonlinear amplifier stage, whereas $K_{r}\left(f\right)$ determines the effective rectification efficiency and the resulting DC polarity. The susceptible frequencies used in this work, such as 198 MHz, were determined by frequency sweeping under a fixed excitation level. During the sweep, the DC component of $V_{\mathrm{amp},\mathrm{out}}$ was recorded at each frequency point, and frequencies producing obvious and repeatable positive or negative DC offsets were identified as susceptible frequencies.

The comparator is triggered when the nominal amplifier output plus the RF-induced DC offset at a susceptible frequency exceeds the reference threshold:

(8) $$V_{\mathrm{amp},\mathrm{out},0}+\Delta V_{\mathrm{amp},\mathrm{out}}\left(f\right)\geq V_{\mathrm{ref}},$$

where $V_{\mathrm{amp},\mathrm{out},0}$ is the nominal amplifier output without RF injection and $V_{\mathrm{ref}}$ is the comparator reference threshold. This condition links the frequency-dependent RF-induced output offset to the final false-triggering behavior of the smoke detector.

Consequently, signal injection at the amplifier’s input stage can effectively deceive the smoke detector into registering a phantom smoke event. This vulnerability is experimentally validated in the following section.

### 4.2. Experimental Verification on Smoke Sensing Circuitry

To verify the previous analysis, we conducted feasibility tests to explore the capability of IEMI to affect smoke sensors [15].

We used an Arduino UNO to read the output analog voltage every second and sent the data to a PC through the serial port. To prevent the Arduino from being affected by IEMI, we wrapped it with electromagnetic shielding material. All experimental components are readily available on the market.

**Figure 9:**
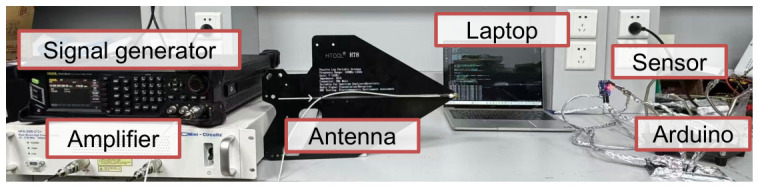
Experimental setup of the smoke sensor module, illustrating the equipment configuration for targeted EMI injection testing.

Subsequently, we used an EXG vector signal generator [16] to generate a 100MHz∼1GHz signal, used an HPA-25W-272+ amplifier [17] to amplify it to 10W, and emitted it through a directional antenna [18] with a gain of +12dBi at a distance of 60cm. The experimental setup is shown in Figure 9.

**Figure 10:**
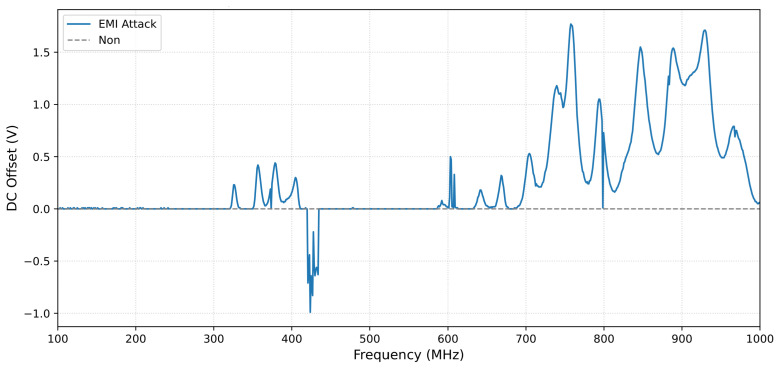
Experimental results of the frequency sweep tests show that, at specific interference frequencies, the analog output signal of the sensor exhibits deviations, including both positive and negative offsets.

We conducted frequency sweep tests to evaluate the sensor’s vulnerability. As illustrated in Figure 10, the results demonstrate that the sensor’s output can be artificially increased or decreased at specific interference frequencies, which confirms our theoretical analysis of both the rectification and amplification effects.

To verify whether the output of the smoke sensor module can be stably and precisely controlled, we selected two sensitive frequency points that induce positive and negative offsets: 760 MHz and 420 MHz, respectively. The injected EMI signal can be modeled as a sinusoidal carrier signal:

(9) s(t)=c(t)=Asin(2πft),

where *A* denotes the signal amplitude, *f* represents the interference frequency, and *t* is time. By selecting different sensitive frequencies, the injected signal can induce different offset directions at the sensor output.

Furthermore, considering the signal transmission process, the power variation over the air can be calculated using a simplified version of the Friis transmission equation:

(10) $$P_{r}=P_{t}G_{t}G_{r}{\left(\frac{\lambda }{4\pi d}\right)}^{2}$$

where λ is the signal wavelength, and *d* represents the attack distance. $P_{r}$ and $P_{t}$ denote the received and transmitted power, respectively. $G_{t}$ and $G_{r}$ represent the gains of the transmitting and receiving antennas, which inherently account for the effects of the radiation angle.

Assuming the attacker has previously profiled the injection at a baseline distance $d_{0}$ with a transmit power $P_{t0}$, she can adjust the transmit power to maintain the identical attack effect at a new distance *d* using the following proportional relationship:

(11) $$P_{t}={\left(\frac{d}{d_{0}}\right)}^{2}P_{t0}$$

**Figure 11:**
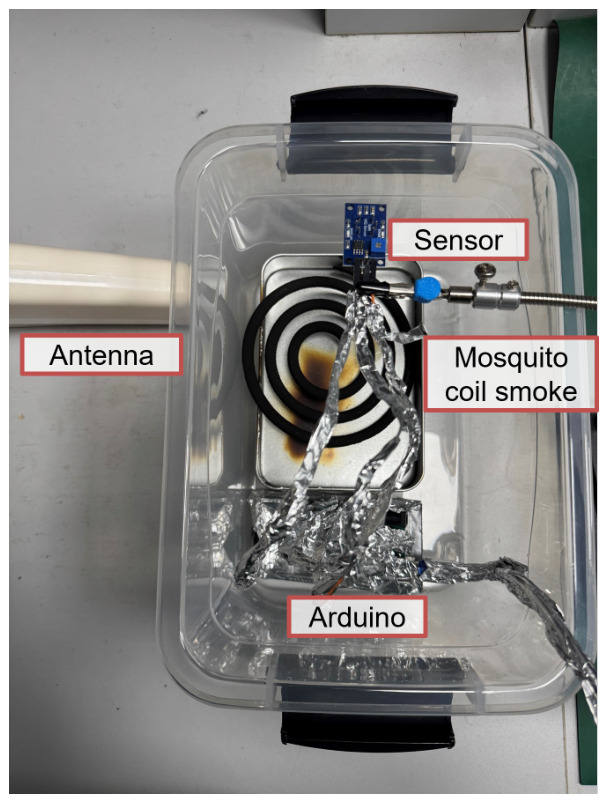
Setup for experiments under smoke conditions.

**Figure 12:**
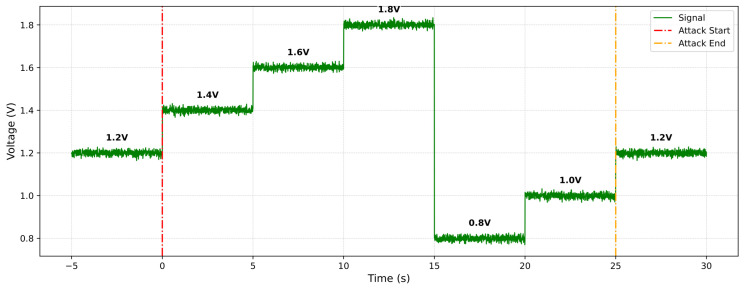
Experimental waveforms illustrating the induced positive and negative DC offsets under varying transmission power levels and specific resonant frequencies.

By dynamically adjusting the signal amplitude *A* according to this model, or by changing the distance between the antenna and the sensor, we can successfully induce specific and controllable DC offsets at the sensor’s output. Under a slightly smoky experimental setup, as shown in Figure 11, the resulting output waveforms demonstrating these targeted deviation attacks are illustrated in Figure 12. In practical attack scenarios against smoke detectors, increasing the sensor’s output value beyond the comparator’s threshold can trigger a false alarm, whereas decreasing the output in the presence of actual smoke can effectively suppress the alarm.

### 4.3. The Impact of IEMI on Button Circuits

**Figure 13:**
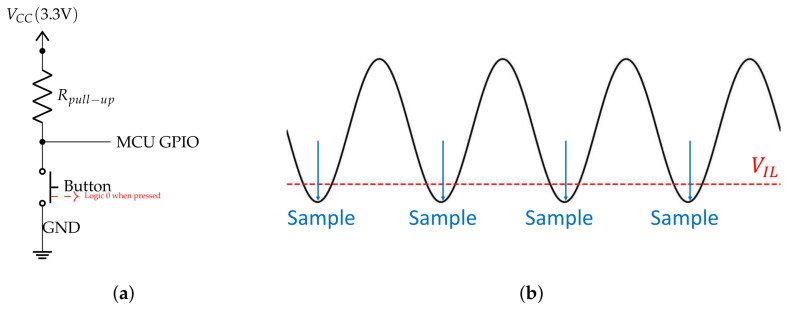
Button circuit and sampling results: (**a**) schematic diagram of the button circuit with a pull-up resistor, and (**b**) sampling results of the button signal.

To induce a valid button event through IEMI, the disturbance must satisfy the basic button-detection logic of the target device. In a typical pull-up button circuit, as shown in Figure 13a, the input pin remains at a high idle voltage when the button is released. When the button is pressed, the input node is pulled down. Therefore, the MCU recognizes the input as a button press only when the input voltage falls below the logic-low threshold $V_{IL}$:

(12) $$V_{in}\left(t\right)\leq V_{IL}.$$

However, a momentary threshold crossing is usually insufficient to generate a valid button event. In practical systems, the MCU periodically samples the button input and applies software debouncing to filter out transient glitches and mechanical contact bounce. Thus, the input must be detected as logic low over multiple consecutive sampling cycles:

(13) $$V_{in}\left(t_{k}\right)\leq V_{IL},\;k=1,2,\ldots ,N,$$

where $t_{k}$ denotes the MCU sampling instants, and *N* represents the number of consecutive low-level samples required by the debouncing logic, as shown in Figure 13b. For commercial smoke detectors, however, the firmware implementation is generally inaccessible to end users. Therefore, the effective polling rate, debounce algorithm, and the exact number of consecutive samples required for event validation cannot be directly obtained from the tested devices. In this work, $t_{k}$ and *N* are therefore used to describe a general button-detection model rather than known firmware parameters of a specific product.

If the IEMI-coupled disturbance at the button input is approximated as a sinusoidal signal, the effective input voltage can be modeled as:

(14) $$V_{in}\left(t\right)=V_{Idle}-A\sin\left(2\pi ft+\varphi \right),$$

where $V_{Idle}$ is the idle voltage of the pull-up input, *A* is the equivalent amplitude of the coupled disturbance, *f* is the disturbance frequency, and φ is the initial phase. In this case, the MCU samples the input as logic low only when the sampled voltage satisfies:

(15) $$V_{Idle}-A\sin\left(2\pi ft_{k}+\varphi \right)\leq V_{IL},\;k=1,2,\ldots ,N.$$

This equation demonstrates that the high-frequency disturbance must not only have sufficient amplitude to pull the input voltage below $V_{IL}$, but also maintain this condition at the MCU sampling instants. In an idealized sampling model, one possible condition that can increase the probability of repeated low-level samples is phase alignment between the disturbance waveform and the MCU sampling instants. For example, if the disturbance frequency is close to an integer multiple of an assumed sampling frequency $f_{s}$, i.e., $f\approx m\cdot f_{s}$, where *m* is a positive integer, the sampled phase may remain relatively stable over consecutive sampling cycles. Under such a condition, once the sampled waveform falls near a trough and the input voltage is pulled below $V_{IL}$, subsequent samples may also observe a low-level state, thereby satisfying the debounce logic.

Nevertheless, this integer-multiple relationship should not be interpreted as a strict requirement for the tested commercial smoke detector. Since the actual firmware-level polling rate and debounce parameters are unknown, we cannot claim that the selected interference frequency is exactly synchronized with the MCU sampling frequency. Instead, the key requirement is that the IEMI-induced low-level disturbance persists or recurs within the device’s effective sampling and debounce window. The sensitive-frequency condition was therefore identified empirically through repeated frequency-sweep and exposure experiments, rather than calculated from disclosed firmware parameters.

**Figure 14:**
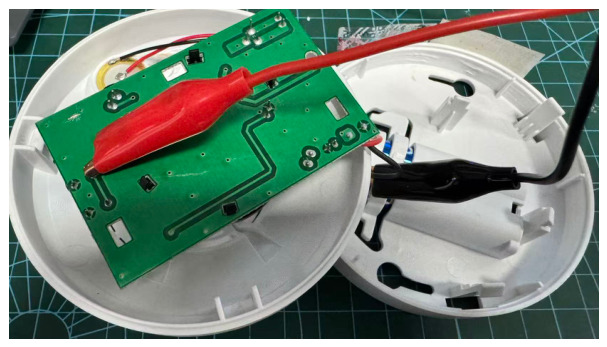
Experimental setup for wired injection on the button.

Based on the above analysis, we performed a wired injection experiment by setting the output power level to 0 dBm and the frequency to 200 MHz. Button-event injection was successfully achieved on a commercial smoke detector, model RFY-831, as shown in Figure 14. The successful triggering result indicates that the induced disturbance can survive the black-box sampling and debounce process of the device under the tested condition. However, because the internal firmware is inaccessible, this result should be interpreted as an empirical validation of button-event susceptibility rather than a direct measurement of the device’s polling rate or debounce sample count.

In summary, by injecting a button-event signal into the circuit, both false alarms and alarm suppression can also be achieved. This result further shows that IEMI can affect not only the analog sensing path, but also the user-interface input path. Due to the black-box nature of commercial firmware, the exact software debounce parameters remain unknown; nevertheless, the repeated triggering behavior under the identified test condition demonstrates that the injected disturbance can be accepted by the device as a valid button event.

## 5. Evaluation

**Figure 15:**
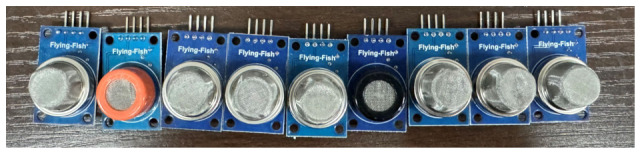
Different sensor modules tested in the experiments.

**Figure 16:**
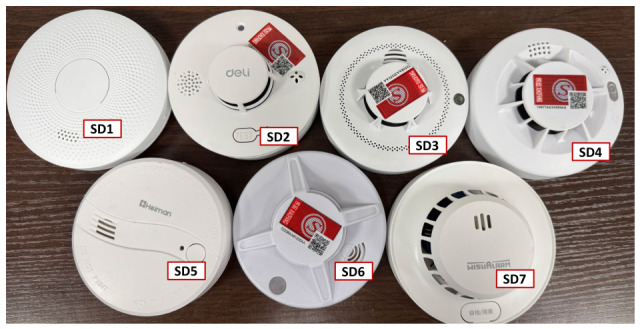
Photograph of the commercial smoke detector tested in the experiment.

In this section, we evaluate this attack on 9 smoke sensor modules and 7 commercial smoke detectors across different manufacturers and structures, as shown in Figure 15 and Figure 16. The device model information of the tested commercial smoke detectors and sensor modules has been provided in the corresponding tables.

Unless otherwise specified, the IEMI signal was generated by the same EXG vector signal generator used in the previous feasibility experiments. The amplifier followed the same configuration as described earlier and did not require additional adjustment. The signal was radiated toward the target device through a directional antenna with a gain of approximately +12 dBi.

Based on prior experimental experience, we selected the following frequency ranges for the frequency-sweep experiments: the commercial smoke detectors were swept over the frequency range from 600 MHz to 2.5 GHz with a step size of 1 MHz, while the smoke sensor modules were swept over the frequency range from 100 MHz to 1 GHz with a step size of 1 MHz. The sweep interval at each frequency point was set to 1 s. At each tested frequency point or spatial configuration, the detector response was monitored within a 30 s observation window to determine whether the expected alarm manipulation occurred.

We then explore the factors affecting injection performance, including the antenna angle, injection distance, and obstacles. Meanwhile, we validated the feasibility of this attack in a real-world scenario, where a commercial smoke detector was installed on the actual ceiling of an ordinary room.

For smoke-present experiments, we used a small-scale and controllable smoke source, such as a mosquito coil or cigarette, to create a reproducible smoke condition. The smoke-present experiments were conducted in an ordinary plastic storage box, where the detector and the smoke source were placed at relatively fixed positions in each trial to maintain consistent experimental conditions. After each trial, the detector state was reset and the plastic storage box was ventilated before the next measurement.

We further evaluated the smoke-present alarm suppression effect under low, medium, and high smoke levels, and the results show that IEMI delayed the detector alarm across all tested smoke levels, while denser smoke reduced the suppression duration.

Finally, we conducted experiments on several common gas detectors to demonstrate the generalizability of the proposed attack.

### 5.1. Different Smoke Detectors and Modules

The overall performance of each smoke detector is summarized in Table 1. In the table, false-alarm sensitive frequency denotes the frequency at which IEMI caused the detector to generate an alarm in the absence of smoke, whereas alarm-suppression sensitive frequency denotes the frequency at which IEMI prevented the detector from alarming in the presence of smoke. Multiple frequencies indicate multiple sensitive peaks observed during the frequency sweep. The symbol “/” indicates that the corresponding alarm-suppression experiment was not performed for that device due to personal-safety, ethical, and risk-control considerations. Detection Period refers to the time required for each smoke detection cycle, which is typically the period at which the photodiode emits pulsed light. Alarm Decision Logic indicates whether one abnormal sensing cycle was sufficient to trigger the alarm decision or whether multiple abnormal sensing cycles were required for confirmation.

The sensitive frequencies in Table 1 should be interpreted as device-specific coupling peaks rather than universal frequencies. A possible explanation is that the PCB traces, sensor leads, battery wires, power/ground network, and enclosure openings of different detectors may behave as unintended receiving structures at VHF/UHF frequencies. When their effective electrical lengths are comparable to a fraction of the incident wavelength, such as a quarter wavelength or half wavelength, the coupled RF voltage at the analog front-end may be enhanced. For example, frequencies around 800 MHz–1.5 GHz correspond to quarter-wavelength scales of approximately 5–10 cm in free space, which are comparable to typical internal wiring, PCB trace, and enclosure-slot dimensions in compact smoke detectors. Therefore, the observed sensitive frequencies may result from the combined effects of PCB geometry, trace routing, parasitic capacitance, enclosure leakage, and the frequency-dependent response of the analog front-end.

Similarly, we conducted relevant tests on 9 gas sensor modules and recorded their susceptible frequencies along with the maximum offsets. Detailed results are presented in Table 2. By referring to existing standards on environmental pollution and occupational exposure for different types of gases [19,20,21,22,23,24,25,26], we classify gas pollution levels into three basic tiers: safe, mild pollution, and severe pollution. The “safe” tier represents the baseline level under normal environmental conditions. “Mild pollution” indicates a pollution level at which prolonged exposure may cause physical symptoms such as headaches and dizziness. “Severe pollution” refers to a critical hazardous condition in which even short-term exposure may be life-threatening. It can be observed that, after the attacks, the sensor readings can reach the levels of mild or even severe pollution, thereby demonstrating the effectiveness of the attack.

### 5.2. Environmental Factors

**Figure 17:**
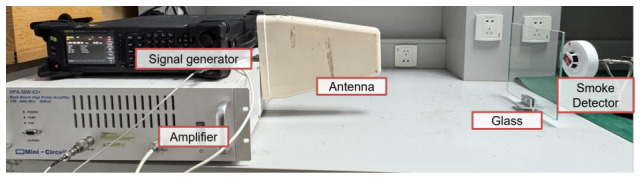
Experimental setup for environmental factor effects.

To quantify the impact of antenna position, injection distance, and obstacles on the effectiveness of this attack, we conducted experiments using an SA-Y-1 smoke detector, with the experimental setup shown in Figure 17. The only difference is that, during the obstacle experiments, an obstacle was placed between the target detector and the antenna to evaluate the impact of its presence on the attack performance.

#### 5.2.1. Position and Distance

**Figure 18:**
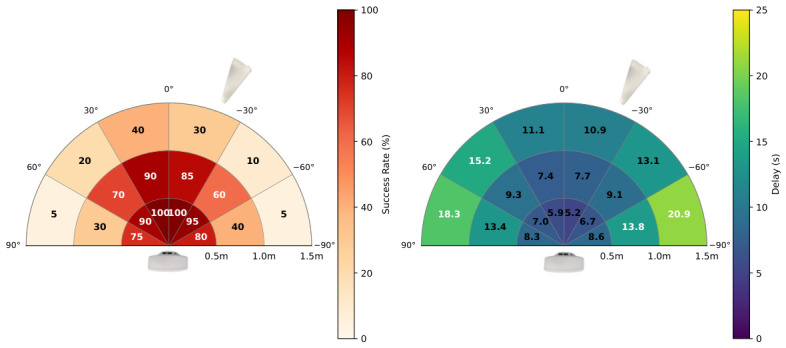
Average success rate and attack delay at different distances and angles.

In our experiments, the distances between the antenna and the smoke detector were set to 50 cm, 100 cm, and 150 cm. We also evaluated the impact of the injection angle across a sector at 30° intervals. At each specific position and angle, we performed 20 independent repeated trials, using the average time required for a successful attack and the success rate (i.e., whether the attack succeeded within 30 s) as metrics for evaluating attack effectiveness. The results are summarized in Figure 18.

As can be observed, although the average attack time increases and the average success rate decreases with greater distances and angular deviations, the attack can still be successfully carried out under most test conditions. This demonstrates the robustness and effectiveness of the proposed attack, which can be performed over long distances without stringent angular requirements. In practical experiments, we successfully induced false alarms in the smoke detector at a distance of 3.6 m.

#### 5.2.2. Obstacles

**Figure 19:**
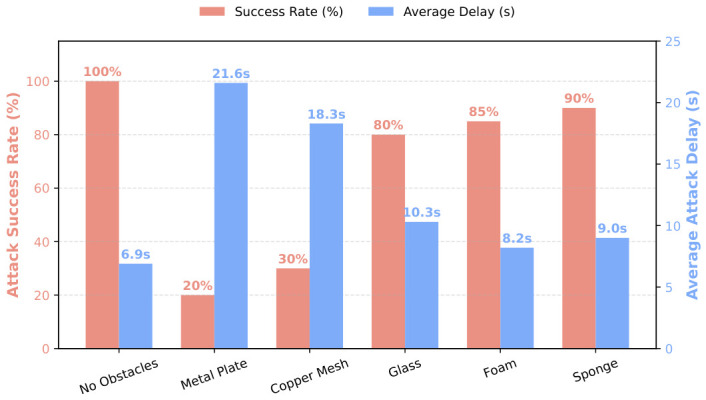
Effect of obstacles on attack performance.

In practical attack scenarios, an adversary may not be physically present in the room where the smoke detector is installed and may only be able to launch the attack through a window or a wall. In this section, we analyze the impact of different obstacles on the effectiveness of the attack. A total of five types of obstacles were considered in the experiments, including Metal Plate, Copper Mesh, Glass, Foam, and Sponge. For each type of obstacle, 20 trials were conducted at a distance of 50 cm. The results are shown in Figure 19. It can be observed that the effectiveness of the attack is significantly reduced in the presence of a metal plate or copper mesh, whereas the attack remains effective under other conditions. Additionally, since smoke detectors are typically deployed in relatively open spaces, this provides multiple angles and entry points for launching the attack.

### 5.3. Smoke-Present Alarm Suppression Evaluation

To further evaluate the alarm suppression effect in the presence of smoke, we conducted controlled experiments at three experimentally defined smoke levels: low, medium, and high. All experiments were performed in a fixed-size plastic chamber, with the positions of the smoke detector and smoke source kept unchanged. Because calibrated optical obscuration and particulate-matter measurements were unavailable, the smoke level was quantified using the average rate of increase in the estimated smoke concentration over a 10 s interval. The smoke concentration was estimated from the output voltage of the MQ-2 sensor using the voltage-to-concentration conversion adopted in our experimental setup. The low, medium, and high smoke levels corresponded to average concentration increase rates of approximately 56.4 ppm/s, 161.1 ppm/s, and 364.8 ppm/s, respectively. Although this sensor-based metric does not replace a calibrated smoke concentration measurement, it provides a measurable and reproducible approximation of the smoke conditions used in this study.

For each smoke level, experiments were conducted both without and with IEMI. In the experiments without IEMI, the baseline alarm time was measured as the interval between the start of smoke exposure and the first alarm generated by the detector. In the experiments with IEMI, the alarm time was measured under the corresponding smoke condition. Smoke was generated independently in each trial, and each experimental condition was repeated 10 times.

The alarm suppression duration was defined as the additional alarm delay caused by IEMI relative to the baseline condition at the corresponding smoke level. For each IEMI trial, it was calculated by subtracting the mean baseline alarm time for the corresponding smoke level from the observed alarm time under IEMI. The baseline alarm time and alarm suppression duration are reported as the mean ± standard deviation over 10 repeated trials.

As shown in Table 3, the mean baseline alarm time decreased from 28.7±8.7 s at the low smoke level to 9.4±3.2 s at the high smoke level, indicating that the detector responded more quickly as the smoke concentration increased more rapidly. Under IEMI, the mean alarm suppression durations were 27.4±6.4 s, 15.3±4.2 s, and 10.4±3.1 s at the low, medium, and high smoke levels, respectively. A positive suppression duration indicates that IEMI delayed the alarm relative to the corresponding baseline condition. The results therefore show that IEMI delayed smoke-triggered alarms at all three tested smoke levels.

The alarm suppression duration decreased as the smoke concentration increase rate rose, suggesting that a more rapidly increasing smoke concentration may reduce the alarm suppression effect of IEMI. However, because smoke was generated independently in each trial and the smoke level was characterized using an approximate sensor-based metric, this trend should be interpreted only within the current experimental setup and the tested smoke range.

### 5.4. Attack in a Real-World Scenario

**Figure 20:**
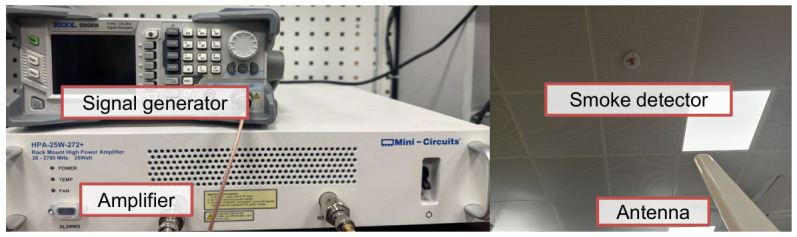
Attack in a real-world scenario.

To further evaluate the feasibility of the attack under conditions closer to practical deployment, we conducted an experiment in an ordinary indoor room, where a commercial smoke detector was installed at a typical ceiling-mounted position, as shown in Figure 20. It should be noted that the term “real-world scenario” in this paper refers to a controlled indoor experimental configuration that more closely resembles practical installation, rather than a comprehensive validation across different building structures, construction materials, and deployment environments.

Based on the preceding laboratory tests, a real-world attack would generally require prior identification of the target device model and frequency-sweep testing to determine key parameters, such as the sensitive frequencies, modulation settings, and transmission power. Because different devices may employ different circuit structures and alarm logic, parameters obtained for one model may not directly apply to another.

After determining the attack parameters, the attacker would also need to select a favorable antenna orientation and a relatively short attack distance to improve electromagnetic coupling and increase the success rate. Walls, metal structures, and other obstacles should be avoided whenever possible, as they may introduce attenuation, reflection, and blockage.

Therefore, practical execution depends on target identification, device-specific profiling, favorable antenna placement, and a sufficiently unobstructed propagation path. The experiment presented in this subsection demonstrates feasibility when these conditions are reasonably satisfied, rather than indicating that the attack can be reliably performed against arbitrary devices or in arbitrary building environments.

### 5.5. Gas Monitor

In addition to smoke detectors in fire protection systems, adversaries could also affect systems equipped with other classes of gas sensors by exploiting a similar hardware-level vulnerability. The key reason is that these devices commonly rely on analog front-end amplifiers to process weak sensor-level signals. Once the injected electromagnetic interference is coupled into the sensing path or the analog front-end input, the disturbance may be amplified together with the legitimate sensor signal and then propagated to subsequent comparison, digitization, display, or alarm-decision stages. Therefore, the vulnerability is not limited to a specific sensing material or target gas, but is closely related to the shared signal-conditioning architecture, especially the amplification stage.

Specifically, the alcohol detector relies on a gas-sensitive element whose electrical response changes when alcohol vapor is present; this weak sensor response is then converted and amplified by the analog front-end before being compared, displayed, or used for alarm decision-making. The toxic gas detector follows a similar sensing chain, where the gas-sensitive cell or semiconductor sensing element produces a concentration-dependent weak electrical signal that must be amplified, filtered, and compared by the analog front-end circuits. The air-quality monitor also contains sensing modules whose small analog outputs, such as resistance, current, or voltage variations associated with gas concentration or particulate sensing, are amplified and conditioned before being digitized or interpreted by the control unit. Therefore, although their sensing materials and target gases differ, these devices share a common hardware structure: weak sensor-level signals are first amplified and conditioned by an analog front-end and then passed to a digital decision or display stage.

To avoid treating these experiments as isolated anecdotes, we applied the same evaluation procedure to all three devices. Before IEMI exposure, each device was powered on, allowed to stabilize, and calibrated according to its normal user-level calibration or baseline procedure when available. The baseline reading or alarm state was recorded under normal conditions. We then performed a controlled frequency sweep to identify the sensitive frequency bands of each device, while keeping the device position and test configuration fixed. After identifying the sensitive frequencies, we repeated the exposure at these frequencies under different power levels and monitored whether the displayed value, alarm state, or warning output changed consistently.

**Figure 21:**
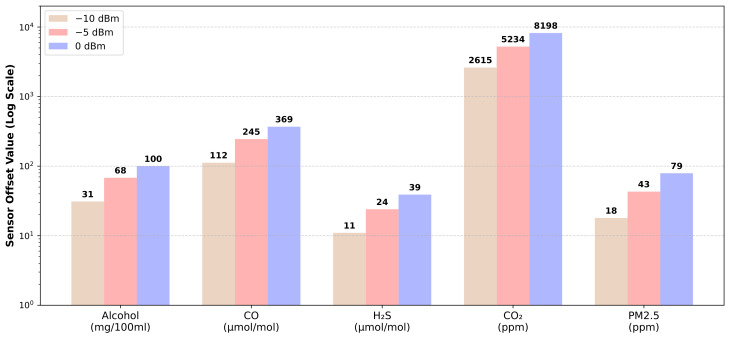
The absolute offset values of various gas and particulate detectors under EMI attacks at power levels of −10 dBm, −5 dBm, and 0 dBm.

**Figure 22:**
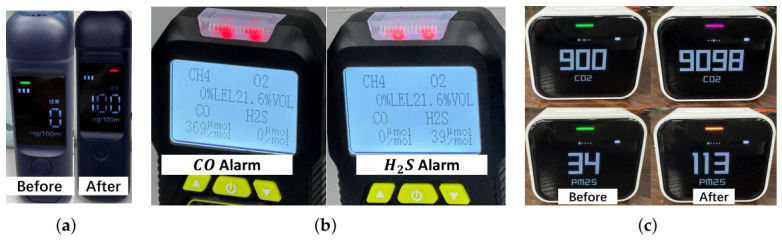
Experiments on commercial gas detectors. (**a**) Alcohol detector. (**b**) Toxic gas detector. (**c**) Air-quality detector.

Following the methodology described in the previous sections, we conducted frequency sweep tests on an alcohol detector, a toxic gas detector, and an air-quality monitor to identify their sensitive frequencies. Subsequently, while maintaining a constant distance, we launched attacks at these specific frequencies using different power levels. For each device, the observed abnormal response appeared repeatedly under the same sensitive-frequency condition and disappeared or weakened when the interference was removed or detuned, indicating that the effect was not caused by random environmental fluctuation. These results support the broader relevance of the proposed mechanism, while we conservatively interpret them as supporting evidence rather than the central contribution of the paper. The experimental results are shown in Figure 21 and Figure 22.

#### 5.5.1. Alcohol Detectors

Alcohol detectors, commonly known as breathalyzers, are electronic devices designed to estimate a person’s blood alcohol content (BAC) by analyzing a breath sample. They are primarily utilized by law enforcement, industrial workplaces, and medical facilities to rapidly assess intoxication levels and prevent alcohol-related accidents. At an attack distance of 0.5 m and an attack frequency of 800 MHz, we were able to force the reading of the alcohol detector to exceed 80 mg/100mL. This manipulation effectively shifts a normal, sober baseline measurement to a level that meets the legal criteria for driving under the influence (DUI) or even driving while intoxicated (DWI). By exploiting this vulnerability, adversaries might be able to maliciously spoof alcohol detection values, thereby creating significant challenges for proper law enforcement.

#### 5.5.2. Toxic Gas Sensor

The detection of toxic gases plays a crucial role in industrial safety, environmental monitoring, and critical infrastructure. These types of sensors are typically integrated into safety-critical systems or SCADA systems. If their analog signals are disrupted or maliciously manipulated, it could lead to catastrophic physical consequences. At an attack distance of 0.5 m, using attack frequencies of 996 MHz and 810 MHz, respectively, we were able to force the measured values of carbon monoxide (CO) and hydrogen sulfide (H_2_S) on a hazardous gas detector to exceed their alarm thresholds. In practical industrial production environments, such manipulations could lead to severe consequences, including human casualties and significant property damage.

#### 5.5.3. Air-Quality Detector

Air-quality detectors are widely deployed in smart buildings, industrial facilities, and cleanrooms to continuously track airborne pollutants such as particulate matter (PM) and volatile organic compounds (VOCs). They play a critical role in automating HVAC systems, ensuring occupational health, and maintaining strict environmental safety standards. For instance, at an attack frequency of 771 MHz, we were able to manipulate the carbon dioxide (CO_2_) concentration reading, causing it to surge from a normal baseline of 900 ppm to 9098 ppm. Furthermore, at an attack frequency of 841 MHz, we successfully increased the PM2.5 concentration from 34 μg/m^3^ to a level that triggered the device’s alarm.

## 6. Countermeasures

Typically, manufacturers install filters to mitigate both external and internal EMI; for instance, common-mode or differential-mode filters are often integrated at the amplifier input. However, as demonstrated in our study, out-of-band IEMI can induce AC signals that bypass conventional filtering and undergo internal rectification via the amplifier’s input, output, or power-supply pins.

While IEMI defense mechanisms are well-established and have been deployed in certain critical sectors, consumer electronics remain notably vulnerable to malicious attacks targeting smoke sensors. In this section, we discuss and simulate several passive and active strategies designed to detect or prevent the adverse effects of EMI on smoke detectors.

### 6.1. Shielding

To prevent long leads from acting as antennas and picking up interference, a standard design practice involves using short shielded wires between smoke sensors and amplifier inputs. However, common-mode noise induced by these “antennas” can convert into differential-mode noise at the circuit connection points. This conversion is driven by the mismatch between the terminal impedance of the cable and that of the receiver circuit. One mitigation strategy involves adding terminating resistors at the contact points to balance these impedances.

While IEMI enclosures can effectively block external interference, they often require apertures for switches, connectors, status indicators, or ventilation. These openings can compromise shielding effectiveness by creating paths for high-frequency interference to penetrate the circuit board. Furthermore, implementing such enclosures necessitates precise thermal modeling of the system.

In scenarios where the smoke sensor must be externally exposed, localized sensor shielding may be employed. This approach is only effective if the shield provides a low-impedance path to ground. However, certain data acquisition systems require the smoke sensor itself to be grounded, similar to specialized probes used in industrial environments. When both the shield and the smoke sensor are grounded, potential differences between the sensor ground and the amplifier ground can trigger ground-loop currents at the input terminals. As IEMI induces common-mode noise, it may propagate through the shield’s ground, creating a loop current that facilitates the rectification effect. While techniques such as placing the shield and sensor ground connections in close proximity, or grounding the shield only at the amplifier end, can reduce this phenomenon, they cannot entirely eliminate it.

**Figure 23:**
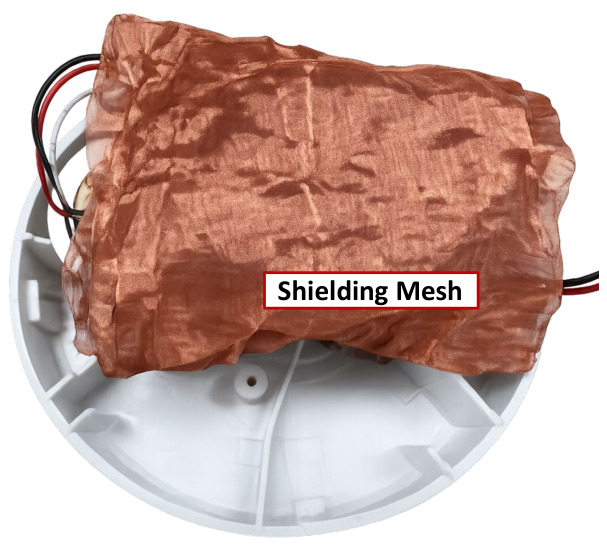
Experimental setup under the shielding condition.

To evaluate the effectiveness of shielding as a practical defense, we wrapped the PCB of the smoke detector with several layers of copper EMI shielding mesh, as shown in Figure 23. To ensure statistical significance, we conducted 20 trials for both the unshielded and shielded configurations. Without shielding, the absolute baseline attack success rate was 95%. After applying the shielding mesh, the residual attack success rate dropped significantly to 25%, representing an absolute reduction of 70%. Additionally, the shielding increased the average attack delay from 6.9 s (variance: 6.7) to 18.3 s (variance: 58), resulting in a net increase of 11.4 s.

Importantly, the mesh structure allows smoke particles to pass through without obstructing the normal smoke-sensing path. To quantitatively verify this, we measured the normal smoke detection time before and after applying the mesh. The baseline smoke detection time was 8.4 s; after shielding, it remained statistically comparable at 9.2 s, confirming that the basic alarm function was not impaired.

Considering the material cost of this defense is less than $1, these results indicate that PCB-level EMI shielding provides an effective and low-cost mitigation against EMI-induced manipulation while preserving the normal functionality of the smoke detector.

### 6.2. Filtering

In the design of operational amplifiers (op-amps) and instrumentation amplifiers (in-amps), manufacturers typically integrate low-pass filters at the input pins to attenuate IEMI signal energy from the input lines. In integrated circuit (IC) smoke sensors utilizing an inverting op-amp configuration, a filter capacitor is positioned between resistors of equal value. Conversely, in IC smoke sensors employing a non-inverting op-amp, the filter capacitor is coupled directly to the amplifier input.

Precision instrumentation amplifiers used in specialized smoke detection probes often employ dual low-pass filters to suppress common-mode signals on each input lane, along with a dedicated capacitor to mitigate differential-mode signals between the two input terminals. However, these filters frequently prove insufficient for complete attack mitigation due to line asymmetry and the specific frequency ranges of injected interference. In certain smoke-sensing architectures, this asymmetry is further exacerbated by the use of dissimilar conductors or complex wiring topologies. Consequently, high-precision smoke detection instruments often require supplementary isolation circuits and active low-pass filters at the input terminals to decouple the field-side sensors from the system-side processing circuitry.

Another protective approach involves a composite configuration of instrumentation amplifiers, such as utilizing three in-amps where two are correlated and connected in antiphase. Choke-based filters may also serve as an alternative for input filtering. However, despite their noise suppression capabilities, the magnetic core materials of the inductors can significantly degrade filter performance at high frequencies, leaving the system vulnerable to targeted IEMI injection attacks.

Finally, amplifier outputs must also be hardened against IEMI. Interference injected onto an output line can couple back into the amplifier’s internal stages, where it undergoes rectification and manifests as a DC offset at the output. While RC filters or ferrite beads in series with the output provide simple, cost-effective shielding, output filtering in smoke detection systems is often restricted to line frequencies and their harmonics (50 Hz/60 Hz) to address noise originating from the main power supply. This narrow focus frequently leaves the system exposed to high-frequency, out-of-band malicious signals.

### 6.3. Algorithmic-Based Detection

**Figure 24:**
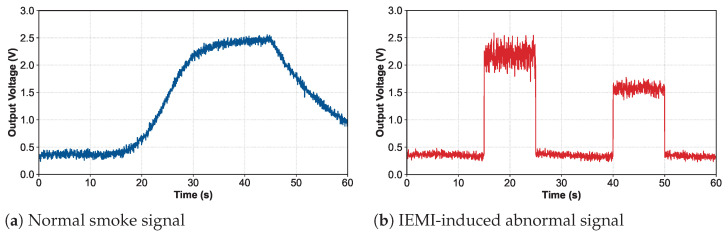
Comparison of smoke detector signals under normal and IEMI conditions.

Under the controlled laboratory conditions evaluated in this study, genuine smoke exposure and IEMI injection produced distinguishable temporal patterns in the collected sensor signals. As illustrated in Figure 24, the smoke-induced signals observed in our experiments generally exhibited smoother temporal variations, whereas the IEMI-induced abnormal readings more frequently showed abrupt and irregular fluctuations. These empirical observations provide preliminary motivation for investigating feature-based software detection methods.

Accordingly, detection algorithms may be implemented on the smoke detector itself or on an associated monitoring unit as a complementary mechanism for identifying potential IEMI-induced abnormalities. However, the current results are limited to the nine tested sensor modules and the controlled experimental conditions considered in this study. Therefore, they do not establish the general robustness or practical deployment readiness of the software-based detector.

Because this study investigates a smoke-detector security threat that has not previously been systematically examined, no publicly available dataset currently exists for this specific scenario. To conduct a preliminary evaluation of the software-based detection approach, we performed a case study using the aforementioned nine sensor modules. An Arduino-based platform was used to collect sensor data under normal operating conditions and multiple IEMI attack conditions, thereby constructing a custom dataset.

To cover several experimental operating conditions, the injection power was set to 5 W, 10 W, and 20 W in the attack scenarios. Both smoke-present and smoke-absent cases were included as normal samples. By configuring the sampling rate, each sample was defined as 10 frames collected within a one-second interval. The resulting dataset contained 2000 samples, including 1000 normal samples and 1000 attack samples. Each sample was assigned a binary label, where “0” denotes a normal state and “1” denotes the presence of an IEMI attack.

**Figure 25:**
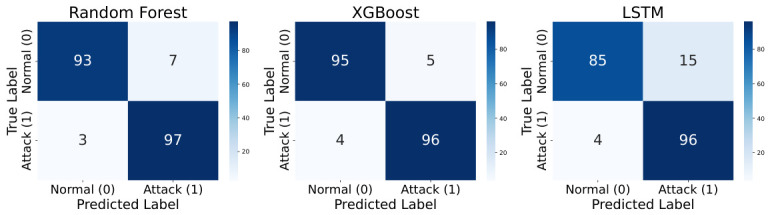
Confusion matrices of different detection models.

The dataset was first divided into training, validation, and testing sets using a ratio of 80%, 10%, and 10%, respectively. We implemented and evaluated several commonly used classification algorithms, including Random Forest, XGBoost, and Long Short-Term Memory (LSTM) networks. Four evaluation metrics—accuracy, precision, recall, and F1-score—were calculated for each model. As reported in Table 4 and illustrated in Figure 25, all three models achieved relatively strong classification performance under the random split setting.

However, because samples from the same sensor module may appear in both the training and testing sets, these results primarily reflect classification performance within the current dataset and may not fully represent the models’ ability to generalize to devices that were unseen during training.

To further investigate whether the software detector can generalize to sensor modules that are not represented in the training data, we additionally conducted a leave-one-device-out evaluation using the existing dataset collected from the nine sensor modules. In each evaluation round, all data collected from one sensor module were excluded from the training process and used only for testing, while data from the remaining eight sensor modules were used for training and validation. This evaluation protocol does not introduce additional data or new detector types, but it provides a stricter assessment of cross-device generalization than the random split setting because the testing module is never observed during model training.

As shown in Table 5, when the testing data were collected from a sensor module excluded from training, the evaluated models still retained some ability to distinguish normal samples from IEMI-induced abnormal samples. However, these results should be interpreted as cross-device performance within the current nine-module dataset rather than as evidence that the software detector possesses general robustness in practical deployment environments.

In this evaluation, Random Forest achieved an accuracy of 95.10%, a precision of 92.03%, a recall of 98.80%, and an F1-score of 95.28%. It also achieved the lowest average false negative rate (FNR) and the lowest worst-case FNR, at 1.20% and 1.80%, respectively. Because false negatives correspond to IEMI attacks that are not detected by the model, these results indicate that Random Forest provided the most favorable FNR performance among the evaluated models under the current experimental conditions. It should be emphasized that this observation is limited to the current dataset and should not be interpreted as demonstrating that Random Forest is universally superior across other detector models or deployment environments.

XGBoost achieved the highest accuracy and F1-score, at 95.30% and 95.39%, respectively. However, its average FNR and worst-case FNR were 2.60% and 5.41%, respectively. Therefore, within the scope of the current evaluation, XGBoost provided a slightly better overall balance across the classification metrics, whereas Random Forest resulted in fewer missed attacks.

LSTM achieved relatively lower accuracy and F1-score values of 86.81% and 87.93%, respectively, while its average FNR and worst-case FNR were 4.40% and 8.11%, respectively. One possible explanation is that the size and diversity of the current dataset are insufficient to support robust sequence-model training under cross-device evaluation. However, larger datasets, hyperparameter analyses, and multiple independent training runs would be required to determine the precise causes of this performance difference.

Overall, the leave-one-device-out results suggest that some of the temporal features used to distinguish smoke-related signals from IEMI-induced abnormal readings may be transferable across the nine tested sensor modules. Nevertheless, these results are limited to the current controlled laboratory conditions and do not demonstrate generalization across different detector models, manufacturers, sensing technologies, or deployment environments. The present study also does not systematically evaluate benign electromagnetic interference, different smoke types and combustion conditions, environmental variations such as temperature, humidity, and airflow, or adaptive attacks that may use low-amplitude, low-rate, or gradually varying signals to evade detection.

In addition, the current experiments were conducted over a limited period and therefore do not characterize sensor aging, calibration drift, false-alarm stability, detection latency, or computational and memory overhead during long-term deployment. Accordingly, the proposed software detector should be regarded as a preliminary and complementary mitigation mechanism rather than a fully validated or deployment-ready defense. Future work will evaluate the approach using a broader range of detector types, benign EMI conditions, different smoke types, adaptive low-rate attacks, environmental variations, and long-term field deployments.

## 7. Discussion

### 7.1. Statistical Robustness and Deployment Variability

**Figure 26:**
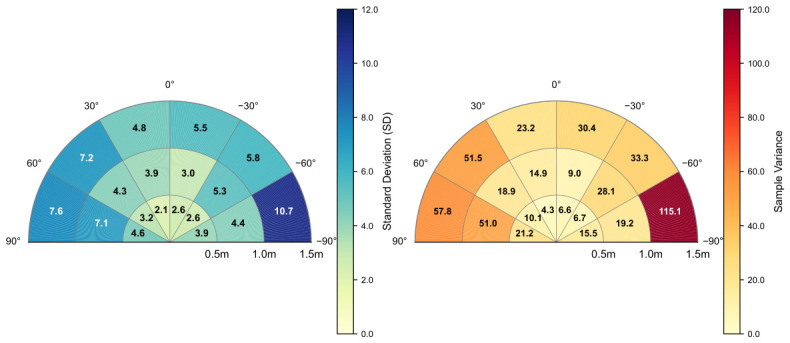
Statistical variability of attack duration under different attack distances and antenna angles. The left panel shows the standard deviation, and the right panel shows the sample variance.

As shown in Figure 26, the attack duration exhibits larger fluctuations under longer attack distances and boundary antenna angles, such as $-90^{\circ }$ and $90^{\circ }$. In contrast, the variations are relatively smaller around the central angular range, especially near $0^{\circ }$. This trend indicates that the attack effect is more stable under favorable electromagnetic coupling conditions, whereas less favorable spatial configurations may increase the uncertainty of the attack duration.

We also considered the negative cases, failure sources, device-to-device variability, and environmental sensitivity observed during the repeated experiments. The unsuccessful trials were mainly associated with abnormal detector power-on status, incorrect device connection, or occasional uncertainties during experimental operation, rather than a systematic failure of the proposed attack mechanism. In repeated tests using detectors of the same model, the device-to-device variation was relatively limited under the same experimental configuration. In addition, within the tested range, changes in battery level, ambient temperature, humidity, and detector placement did not show a substantial influence on the observed attack effect. These observations suggest that the proposed attack phenomenon is repeatable under controlled experimental conditions, while its practical effectiveness may still depend on the quality of the electromagnetic coupling path and the correctness of the experimental setup.

Nevertheless, practical deployment environments may introduce additional uncertainties that are not fully covered by the current controlled laboratory experiments. Factors such as device aging, manufacturing tolerance, long-term battery degradation, environmental variation, and installation differences may affect the internal circuit state, sensing threshold, and electromagnetic coupling path of the detector. Therefore, future large-scale evaluations with more devices, broader environmental conditions, different aging levels, and more diverse installation scenarios are still necessary to further characterize the practical robustness and boundary conditions of the attack.

### 7.2. Portable and Cost-Effective Attack Devices

**Figure 27:**
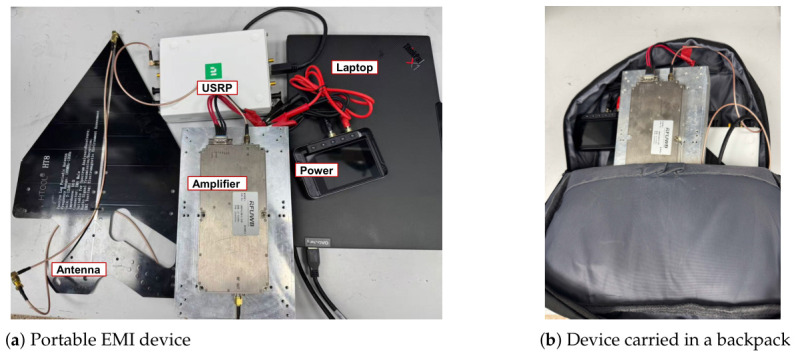
Portable EMI system.

Since previous experimental equipment was typically bulky and expensive, we further evaluated the feasibility of using portable and readily available devices. In our design, we developed a portable electromagnetic interference (EMI) system. We selected a power supply costing $47. We used a USRP B210 [27] as the signal source, with an approximate cost of $2160. We then chose a portable 50 W power amplifier costing $1180. Finally, we used a log-periodic antenna costing $15 to transmit the EMI signal, as shown in Figure 27a. The total cost of the entire system is approximately $3400, and it can be carried in a backpack, as shown in Figure 27b.

### 7.3. Radiated Field Strength Estimation and EMC Context

To further contextualize the reported injection distance and RF exposure level, we added a first-order estimate of the radiated electric-field strength and compared it with laboratory measurements. Under ideal free-space conditions, the approximate RMS electric-field strength at a distance *r* can be expressed as follows [28]:

(16) $$E\left(r\right)\approx \frac{\sqrt{30P_{t}G_{t}}}{r}.$$

Here, E(r) is the estimated RMS electric-field strength, $P_{t}$ is the RF power delivered to the transmitting antenna, $G_{t}$ is the antenna gain in the direction of the target expressed on a linear scale, and *r* is the antenna-to-target distance. The product $P_{t}G_{t}$ corresponds to the equivalent isotropically radiated power in the target direction.

In our experimental configuration, the RF power amplifier had a rated output power of 50 W, and the directional antenna had an approximate gain of +12 dBi, corresponding to $G_{t}\approx 15.85$. Using the rated amplifier output as an approximation of the power delivered to the antenna gives idealized free-space field-strength estimates of approximately 308 V/m at 0.5 m and 51 V/m at 3 m. These values should be interpreted only as first-order reference estimates because the actual power delivered to the antenna was not obtained through a complete measurement of the RF transmission chain.

We also measured the electric-field strength in the laboratory environment. The measured field strength was approximately 131 V/m at 0.5 m and approximately 30 V/m at 3 m. The difference between the measured and calculated values may result from cable and connector losses, antenna mismatch, frequency-dependent antenna gain, non-ideal antenna alignment, polarization mismatch, environmental reflections, and spatial interference. In addition, the simple inverse-distance relationship assumes free-space propagation and a field that can be approximated as a plane wave. The validity of these assumptions depends on the operating wavelength, antenna dimensions, and observation distance and was not independently verified at every tested frequency, particularly at the shorter separation distance. Therefore, the calculated values are treated as idealized free-space reference estimates, whereas the measured values more directly represent the exposure conditions in our laboratory.

The simplified estimate can also be placed in the context of IEC 61000-4-3, which provides a standardized method for evaluating the immunity of electrical and electronic equipment to radiated RF electromagnetic fields [29]. Unlike the above analytical relationship, IEC 61000-4-3 establishes defined test levels and procedures and emphasizes a characterized and reproducible electromagnetic test environment. Thus, the standard evaluates equipment performance under a controlled incident field rather than inferring the field solely from transmitter power, antenna gain, and separation distance.

Our experimental procedure differs from standardized IEC 61000-4-3 radiated-immunity testing in several respects. We did not perform a formal field-uniformity calibration over a standardized test plane, verify the complete immunity-chain linearity according to the standard, apply all prescribed test signals and modulation conditions, or evaluate the detector using standardized performance criteria. Instead, the frequency, antenna orientation, distance, and coupling configuration were intentionally adjusted to identify security-relevant susceptibility and circuit-level coupling paths. Consequently, the results should not be interpreted as evidence that the tested detectors pass or fail IEC 61000-4-3 or any related product-level EMC requirement.

The simplified field-strength relationship should also not be regarded as a comprehensive RF link budget. A complete link-budget or coupling analysis would require the actual frequency-dependent transmitter output, cable and connector losses, antenna mismatch, measured antenna radiation pattern and polarization, propagation and multipath effects, target orientation, effective receiving aperture, enclosure penetration, PCB-level coupling paths, and the frequency-dependent susceptibility of the internal circuits [30]. These factors were not fully characterized in the present study. Accordingly, the analytical expression is used only to provide a transparent first-order relationship among nominal transmit power, antenna gain, distance, and incident field strength.

For additional product-level context, the measured field strengths may be compared with the commonly referenced EN 50130-4 radiated RF immunity level of 10 V/m. The measured field strength of approximately 30 V/m at 3 m was about three times this level, while the measured field strength of approximately 131 V/m at 0.5 m was about thirteen times this level. However, this numerical comparison does not constitute an EN 50130-4 compliance assessment because the standardized test configuration and procedures were not followed. Overall, our experiments should be interpreted as a controlled, security-oriented demonstration of IEMI susceptibility rather than as a formal EMC immunity test or a complete RF propagation and coupling analysis.

## 8. Related Work

In the following, we summarize the existing attacks and defenses utilizing IEMI and Fire Alarm System Security.

IEMI Attacks. Prior works have thoroughly investigated the vulnerabilities of sensors and actuators to IEMI. Foo Kune et al. [31] provided the foundational analysis of IEMI attacks on analog sensors. Since then, the attack surface has expanded significantly, with reported vulnerabilities in microphones [31,32], touchscreens [33,34,35], temperature sensors [36], LiDAR [37], image sensors [38,39], and inverters [40].

As summarized in Table 6, existing EMI attacks mainly target perception sensors and CPS actuators, relying on electromagnetic coupling through analog front-ends or signal lines, and typically achieve either denial-of-service (DoS) or malicious data injection. Our work systematically compares prior attacks across multiple dimensions, including attack target, signal type, attack frequency, amplifier utilization, power, distance, and attack effect. Importantly, the comparison highlights that prior works rarely demonstrate control over safety-critical alarm behaviors, whereas our attack uniquely enables both false alarm triggering and alarm suppression in smoke detectors, and further extends to gas-sensing systems via analog front-end coupling mechanisms.

IEMI Defenses. With the proliferation of IEMI attack methods, defensive techniques are continually evolving. Current research on defense against IEMI includes passive defense based on shielding [43,44,45,46] and filtering [47,48,49], and active defense based on detection. Active defense includes adding extra detection circuits to detect IEMI [50,51,52,53,54], encoding critical signals secretly to detect IEMI [38,55,56], and designing algorithm based on sensor characteristics to detect IEMI [34,57,58,59,60].

Fire Protection System Security. To the best of our knowledge, there is a paucity of academic research addressing the vulnerabilities of smoke detectors integrated into fire alarm systems. Shin et al. [6] conducted a systematic security analysis of OBSDs used in critical infrastructure. They demonstrated that by injecting malicious **optical signals** (e.g., lasers), an attacker could remotely saturate the receiver to induce false alarms (the “cried wolf” effect) or inject synchronized light pulses to suppress real fire alarms. In contrast, our approach primarily focuses on point-type smoke detectors, which are more widely deployed across diverse application scenarios. Furthermore, due to the presence of the optical chamber, prior optical-based attack method is difficult to realize in these systems.

## 9. Conclusions

In this paper, we systematically analyze the security of the commercial smoke detectors and detector architectures evaluated in this study and reveal the threats posed by IEMI to their button and amplifier circuits. This work demonstrates that, under the evaluated laboratory conditions, an attacker can induce false alarms or suppress smoke-triggered alarms in the tested devices without physically touching the target system. We validate the feasibility of the attack through experiments on seven commercial smoke detectors and nine gas sensor modules. We further observe related IEMI-induced effects in the additional commercial gas-detection devices evaluated in this study. In addition, we discuss potential countermeasures against such attacks. These findings should be interpreted within the scope of the devices, detector architectures, and experimental conditions evaluated in this study, rather than as evidence that all smoke detectors exhibit equivalent susceptibility. We hope that this work can raise awareness of the security of critical components in fire protection systems.

## 10. Ethical Considerations

Given that this research involves the manipulation of critical life-safety sensors and fire protection systems, the discovery and analysis of these physical-layer vulnerabilities entail significant ethical responsibilities. The integrity, safety, and ethical soundness of this work were maintained by strictly adhering to the following principles, safety protocols, and responsible handling measures.

### 10.1. Safety Precautions and Laboratory Isolation

To eliminate any potential threat to human life, personal property, or public infrastructure, all physical experiments evaluating Intentional Electromagnetic Interference (IEMI) were conducted exclusively in a highly controlled, isolated laboratory testbed environment. In experimental scenarios where smoke-present conditions were required to validate alarm suppression vulnerabilities, the system was isolated within localized, non-hazardous experimental enclosures using small-scale, transient test smoke sources, such as mosquito coils. Standard and redundant fire-prevention countermeasures were strictly maintained by laboratory personnel throughout the data collection process to ensure experimental safety.

### 10.2. Nature of the Contribution and Defensive Scope

The objective of this study is strictly defensive and investigative. The primary goal is to expose a widespread, underlying hardware-level vulnerability, specifically the out-of-band high-frequency rectification effects in amplifier front-end and button circuitry, before it can be exploited in the wild by malicious actors. The evaluation was strictly limited to commercially available off-the-shelf (COTS) products and standard sensor modules obtained through authorized commercial channels. No personal, third-party, or proprietary infrastructure devices were targeted or disrupted outside the isolated test facility.

### 10.3. Dual-Use Risks and Technical Balance

We explicitly acknowledge the dual-use potential inherent in uncovering these physical flaws, as the described “Invisible Smoke” vulnerability analysis could theoretically be repurposed by adversaries to cause disruptive nuisance alarms or dangerously suppress genuine fire-safety warnings. While our empirical validation demonstrates that such effects can occur under contactless experimental conditions at practical distances and with relatively low aggregate power consumption, replicating the precise control loop requires an understanding of device-specific circuit characteristics and nonlinear signal responses. Therefore, to responsibly balance full vulnerability disclosure against the risk of weaponization, we carefully calibrated the depth of experimental parameters shared in this paper.

### 10.4. Responsible Disclosure and Risk Mitigation

In close alignment with industry-standard responsible disclosure practices, the identified hardware vulnerabilities, empirical data, and technical findings have been compiled and reported to the relevant equipment vendors and product standardization authorities. Furthermore, to prevent direct, unskilled replication or automated misuse of our findings, highly granular and fine-tuned attack execution vectors, such as exact modulation profiles and explicit synchronization sequences required to bypass multi-sample software debouncing algorithms, have been intentionally omitted from this paper as a safety precaution.

### 10.5. Decision to Publish and Anticipated Benefits

The decision to publish is motivated by the critical need to address systemic gaps between conventional electromagnetic compatibility (EMC) testing regulations and actual physical-layer security guarantees in sensor networks. Since current commercial safety detectors are generally audited under compliance standards that primarily target non-malicious environmental noise, the latent vulnerabilities identified in this study may remain unaddressed without proactive academic scrutiny. Publishing these findings provides a scientific foundation that alerts industry stakeholders, incentivizes manufacturers to strengthen hardware-level shielding, and accelerates the integration of standardized, resilient, feature-based detection algorithms to fortify next-generation life-safety infrastructure.

### 10.6. Ethical Approval and Safety Scope

This study did not involve human participants, human biological materials, animals, or the collection of personal data; therefore, formal human-subject ethical approval was not applicable. Nevertheless, because the research involved safety-critical sensing devices and controlled electromagnetic interference experiments, the authors implemented laboratory-level safety and risk-control procedures throughout the study. All experiments were conducted by trained personnel in an isolated laboratory testbed, and no operational fire-protection infrastructure or public safety system was affected. 

## Figures and Tables

**Table 1:** Operating Characteristics and Sensitive Frequencies of Tested Smoke Detectors.

Device Model	False-Alarm Sensitive Freq. (MHz)	Alarm-Suppression Sensitive Freq. (MHz)	Detection Period (s)	Alarm Decision Logic
Hikvision HF-Y3C	800	880	14.32	Single-cycle threshold decision
Dahua S30	1174, 1405	/	8.63	Single-cycle threshold decision
Deli AG200	998	/	12.61	Multi-cycle confirmation
KKQ-718	1579	740	6.51	Single-cycle threshold decision
SA-Y-1	630, 1200	/	8.36	Single-cycle threshold decision
RFY-831	1150, 1273	941	11.15	Multi-cycle confirmation
Heiman HM-625PHS	1237	/	10.35	Multi-cycle confirmation

Note: “/” indicates that the corresponding alarm-suppression experiment was not performed due to personal-safety, ethical, and risk-control considerations. Multiple frequencies indicate multiple sensitive peaks. Single-cycle and multi-cycle indicate whether one or multiple abnormal sensing cycles were required for alarm decision.

**Table 2:** IEMI Testing Results on Gas Sensor Modules.

Module Type	Nominal Detection Range (ppm)	IEMI Sensitive Freq. (MHz)	Altered Sensor Reading (ppm)	Indicated Risk Level
Smoke Gas (MQ-2)	100–10,000	758	613.76	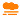
Alcohol (MQ-3)	10–1000	390	51.52	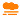
Natural Gas (MQ-4)	300–10,000	164	529.00	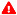
Liquefied Gas (MQ-5)	300–5000	383	659.54	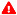
Liquefied Gas (MQ-6)	100–10,000	378	349.95	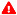
Carbon Monoxide (MQ-7)	10–1000	413	38.73	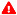
Hydrogen (MQ-8)	50–10,000	332	80.55	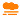
Combustible Gas (MQ-9)	10–1000	917	29.11	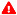
Air Quality (MQ-135)	10–1000	393	26.31	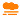

Note: The altered sensor reading denotes the device-indicated output under the tested IEMI condition, rather than an independently calibrated gas concentration. The indicated risk level is used to qualitatively describe the potential consequence of the altered reading: 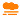 Mild pollution; 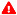 Severe pollution.

**Table 3:** Alarm suppression results under different smoke levels.

Smoke Level	Average Smoke Concentration Increase Rate (ppm/s)	Baseline Alarm Time (s)	Alarm Suppression Duration (s)
Low	56.4	28.7±8.7	27.4±6.4
Medium	161.1	19.1±5.7	15.3±4.2
High	364.8	9.4±3.2	10.4±3.1

**Table 4:** Comparison of Detection Performance across Different Algorithms on the Test Set.

Method	Accuracy (%)	Precision (%)	Recall (%)	F1-Score (%)
Random Forest	95.00	93.27	97.00	95.10
XGBoost	95.50	95.05	96.00	95.52
LSTM	90.50	86.49	96.00	91.00

Note: The reported results were obtained on the held-out test set under the random split setting. Accuracy, precision, recall, and F1-score were used to evaluate classification performance between normal conditions and IEMI attack conditions, where the normal conditions included both smoke-present and smoke-absent cases.

**Table 5:** Leave-one-device-out Evaluation Results of Software-Based IEMI Detection.

Model	Accuracy	Precision	Recall	F1-Score	Average FNR	Worst FNR
Random Forest	95.10%	92.03%	98.80%	95.28%	1.20%	1.80%
XGBoost	95.30%	93.48%	97.40%	95.39%	2.60%	5.41%
LSTM	86.81%	81.52%	95.60%	87.93%	4.40%	8.11%

**Table 6:** Comparison with previous EMI-based sensor attack works.

Work	Attack Target		Attack Method		Attack Capability		
	Victim	Signal Type	Frequency	Use Amplifier	Power (W)	Distance (m)	Effect
[31]	Microphone	Analog	826 MHz	✓	10	1–2	Voice injection
[32]	Microphone	Analog	8–16 GHz	✓	2.5	2.5	Voice injection
[41]	Embedded system	Digital	170–320 MHz	✗	1.8	10	Signal manipulation
[36]	Temperature sensor	Analog	810–950 MHz	✓	3	3	Spoofing
[33]	Touchscreen	Digital	60–90 kHz	✗	6	0.02	False touch
[34]	Touchscreen	Digital	46–86 MHz	✗	/	0.04	Touch injection
[38]	CCD image sensor	Digital	190 MHz	✗	0.1	0.3	Image corruption
[39]	Camera signal line	Digital	1 GHz	✗	/	0.3	Signal distortion
[42]	Servo system	Digital	8–140 MHz	✗	20	0.5	Control disruption
[40]	PV inverter (CPS actuator)	Analog	735–1150 MHz	✓	20	1.5	DoS, control instability & output damping
[37]	LiDAR (ranging sensor)	Analog	500–3000 MHz	✓	50	5	Point cloud jamming, injection & erasure; system crash
Our work	Smoke detector	Analog	630–1405 MHz	✓	50	3.6	False alarm & suppression

Note: ✓ indicates the use of an amplifier, whereas ✗ indicates no amplifier was used.

## Data Availability

The original data presented in the study are included in the article. Further inquiries can be directed to the corresponding author.
